# *Xenopus* Egg Extracts Increase Dynamics of Histone H1 on Sperm Chromatin

**DOI:** 10.1371/journal.pone.0013111

**Published:** 2010-09-29

**Authors:** Benjamin S. Freedman, Kelly E. Miller, Rebecca Heald

**Affiliations:** Molecular and Cell Biology Department, University of California, Berkeley, California, United States of America; Virginia Tech, United States of America

## Abstract

**Background:**

Linker histone H1 has been studied *in vivo* and using reconstituted chromatin, but there have been few systematic studies of the effects of the cellular environment on its function. Due to the presence of many other chromatin factors and specific chaperones such as RanBP7/importin beta that regulate histone H1, linker histones likely function differently *in vivo* than in purified systems.

**Methodology/Principal Findings:**

We have directly compared H1 binding to sperm nuclei in buffer versus *Xenopus* egg extract cytoplasm, and monitored the effects of adding nuclear import chaperones. In buffer, RanBP7 decondenses sperm nuclei, while H1 binds tightly to the chromatin and rescues RanBP7-mediated decondensation. H1 binding is reduced in cytoplasm, and H1 exhibits rapid FRAP dynamics in cytoplasm but not in buffer. RanBP7 decreases H1 binding to chromatin in both buffer and extract but does not significantly affect H1 dynamics in either condition. Importin beta has a lesser effect than RanBP7 on sperm chromatin decondensation and H1 binding, while a combination of RanBP7/importin beta is no more effective than RanBP7 alone. In extracts supplemented with RanBP7, H1 localizes to chromosomal foci, which increase after DNA damage. Unlike somatic H1, the embryonic linker histone H1M binds equally well to chromatin in cytoplasm compared to buffer. Amino-globular and carboxyl terminal domains of H1M bind chromatin comparably to the full-length protein in buffer, but are inhibited ∼10-fold in cytoplasm. High levels of H1 or its truncations distort mitotic chromosomes and block their segregation during anaphase.

**Conclusion/Significance:**

RanBP7 can decondense sperm nuclei and decrease H1 binding, but the rapid dynamics of H1 on chromatin depend on other cytoplasmic factors. Cytoplasm greatly impairs the activity of individual H1 domains, and only the full-length protein can condense chromatin properly. Our findings begin to bridge the gap between purified and *in vivo* chromatin systems.

## Introduction

H1 “linker” histones comprise a highly conserved family of lysine-rich chromatin proteins that promote the folding of beads-on-a-string nucleosome arrays into thicker, 30 nm fibers [1], [2], [3]. Metazoan H1 proteins consist primarily of a winged helix globular domain near the amino terminus and a long, apparently unstructured carboxyl-terminal tail [4]. Nuclease digestion and DNA footprinting experiments suggest a structural model wherein H1′s globular domain localizes near the nucleosome dyad and crosslinks incoming and outgoing DNA, while the tail binds to linker DNA and neutralizes its negative charge [5], [6], [7], [8]. Surprisingly for a structural protein, photobleaching experiments in cells show linker histones to be highly dynamic on chromatin, with residence half-times in the range of seconds to minutes [9], [10], [11], [12]. It is not yet clear how to reconcile these rapid dynamics *in vivo* with the more static view of H1 positioning between nucleosomes that has arisen from work in purified systems [13].

Despite many years of research into histone H1, confusion remains regarding the ability of individual H1 domains to associate with and compact chromatin. Truncated H1 proteins lacking either the globular domain or the unstructured carboxyl terminal tail can have similar effects as full-length H1 on some purified templates [7], [14], [15], [16]. Furthermore, truncated H1 proteins appear to have evolved as bona-fide linker histones in certain unicellular organisms [17], [18]. However, when expressed in vertebrate cells as GFP-tagged fusion proteins, individual domains show severely reduced chromatin binding compared to full-length H1 [10], [11]. A direct comparison between H1 domain function in a purified versus live system might shed light on these apparent contradictions.

The *Xenopus* cell-free system has revealed important information about H1 function in an *in vivo-*like physiological setting. When sperm nuclei, which lack histone H1, are incubated in cytostatic factor (CSF)-arrested metaphase egg extracts, they are remodeled into condensed chromatin, and induce formation of mitotic spindles that appear very similar to those of unfertilized eggs [19], [20]. H1 immunodepletion experiments in egg cytoplasm first identified an essential role for H1 in mitotic chromosome architecture [21]. We recently discovered through substitution experiments that the more negatively-charged embryonic linker histone isoform, H1M (also called B4 or H1oo), binds chromatin with higher affinity than more positively-charged somatic H1A and H10 isoforms, and that phosphorylation of somatic H1 by Cdk1 or phosphomimetic point mutations increase its association with chromatin [22]. This result was surprising, since somatic H1 binds more tightly than H1M to purified dinucleosomes and phosphorylation is believed to weaken H1′s affinity for chromatin [23], [24], again raising the question of whether and why H1 behaves differently *in vivo* than *in vitro.*

Factors that might mediate such differences include RanBP7, which interacts with H1 as a cytoplasmic chaperone and, as a heterodimer with importin beta, a nuclear import receptor [25]. In egg extracts, RanBP7 and importin beta bind specifically to somatic H1 isoforms, but not H1M. Disrupting these interactions with a constitutively active mutant of RanGTP promotes binding of somatic H1 to mitotic chromosomes, suggesting that RanBP7 and importin beta act as cytoplasmic inhibitors of somatic H1 that can be regulated by RanGTP [22]. RanBP7 also interacts with core histones and other basic proteins [26], but it is not known how it affects chromatin. We therefore designed a series of experiments to measure the effect of RanBP7 on H1 binding to sperm nuclei in either buffer or *Xenopus* egg cytoplasm, as well as ability of individual H1 domains to bind chromatin in buffer or extract. We report that cytoplasmic factors including but not limited to RanBP7 and importin beta significantly inhibit the ability of H1 to bind chromatin, and that this inhibition is greater for individual domains of H1 than for the full-length protein. Furthermore, addition of excess H1 or its domains distorts mitotic chromosomes and prevents their segregation during anaphase.

## Results

### RanBP7/Importin beta and Histone H1 Have Opposite Effects on Sperm Chromatin

**Figure 1:**
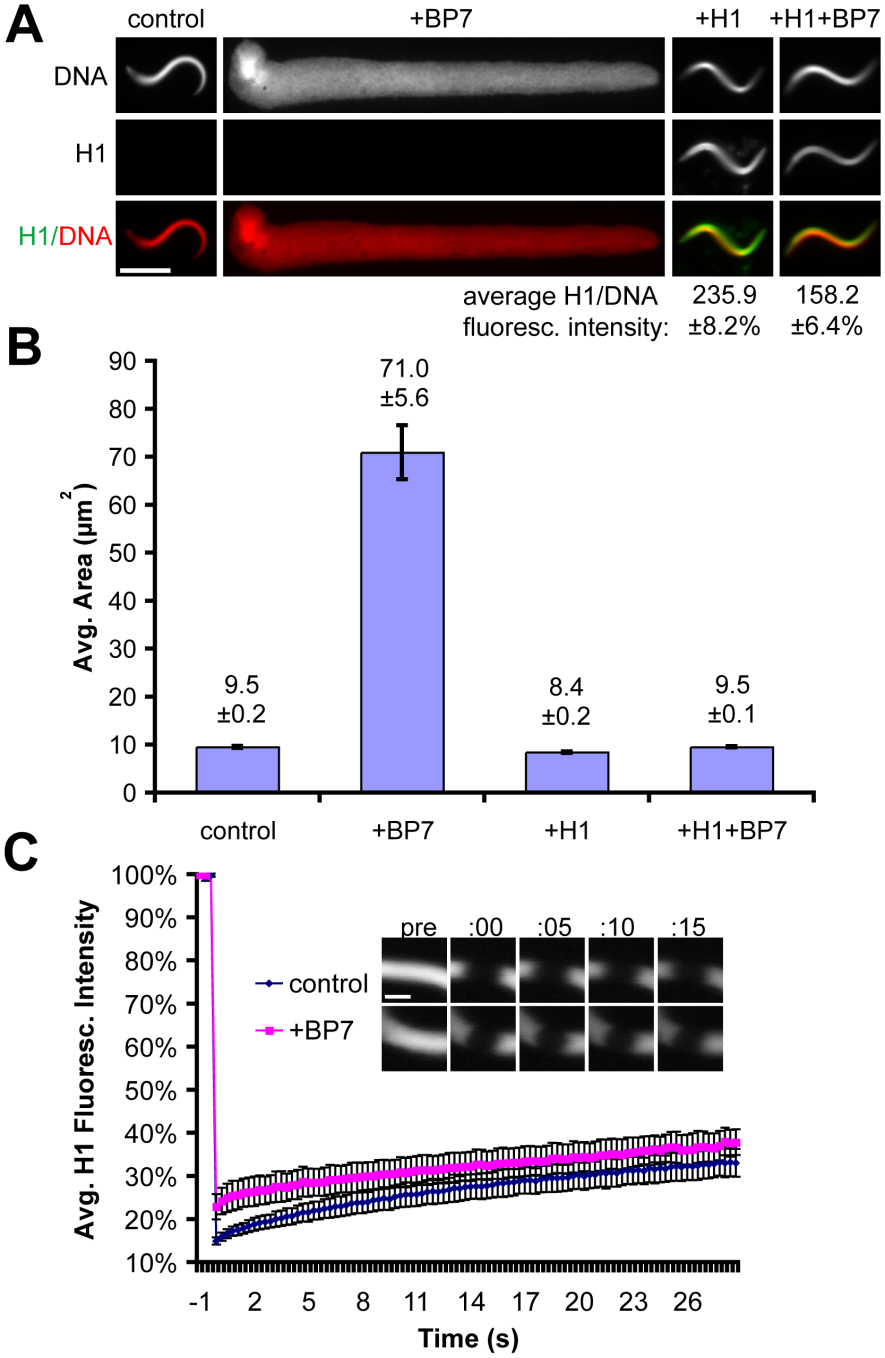
H1 and RanBP7 Have Opposing Activities in Buffer. (A) Fluorescence images of sperm nuclei in buffer with or without 4 µM RanBP7 and 1 µM H1A-GFP. Average H1:DNA fluorescence intensity is shown below for conditions supplemented with H1. Scale bar, 10 µm. (B) Average nuclear area of sperm in buffer (n>50) for conditions described in (A). (C) Averaged FRAP curves (n = 5) and corresponding timelapse images of H1A-GFP on sperm chromatin in buffer with or without RanBP7. The photobleach is plotted at time  = 0. Scale bar, 2 µm. All quantification is shown ± standard error.

First we evaluated the effects of RanBP7 and histone H1 on a simple chromatin template *in vitro*, in extraction buffer (100 mM KCl, 1 mM MgCl_2_, 0.1 mM CaCl_2_, 10 mM K-HEPES pH 7.7, 50 mM sucrose) supplemented with ATP but in the absence of cytoplasm. When mixed with sperm nuclei in buffer, 4 µM RanBP7 caused a dramatic ∼10-fold expansion of sperm chromatin area (Figure 1A–B), similar to the decondensation of sperm nuclei observed in chromatin-assembly extracts or with histone chaperones such as nucleoplasmin [27]. Although RanBP7 had been shown to bind to core histones and other basic proteins [26], this result suggests that import chaperones may also function in the process of sperm chromatin remodeling. We next examined the effect of histone H1 on this system, using the H1A isoform that interacts with RanBP7 in cytoplasm [22], [25]. An H1A-GFP fusion protein was used for this experiment, which has properties similar to somatic H1 [10], [11]. Addition of 1 µM H1 reversed the effects of RanBP7 on sperm area and restored the compact, serpentine nuclear morphology of sperm nuclei even when added after the decondensation had already occurred (Figure 1A–B and data not shown). However, in the presence of RanBP7 the H1:DNA intensity ratio was reduced by approximately 50%, and lower concentrations of H1 between 0.25–0.50 µM could not rescue RanBP7-mediated decondensation, suggesting that this cytoplasmic chaperone competes with chromatin for H1 binding (Figure 1A and data not shown). Since the concentration of sperm base-paired DNA in buffer was ∼4.5 µM, approximately one molecule of H1 was required per 4.5–9.0 base pairs to rescue RanBP7-mediated decondensation of sperm nuclei. This suggests that H1 binds to sperm chromatin in buffer at a ∼30-fold higher stoichiometry than it does *in vivo*, where each H1 molecule binds to a single nucleosome (∼150 bp) [5].

The ability of RanBP7 to decondense sperm nuclei and inhibit H1 raised the possibility that H1 was binding dynamically to chromatin in our system. To test this, we performed Fluorescence Recovery After Photobleaching (FRAP). In contrast to reports in live cells [11], little to no recovery of H1 was observed on sperm chromatin after photobleaching (Figure 1C and Movie S1). Although RanBP7 reduced the localization of H1 to sperm nuclei (Figure 1A), this reduction was not due to an increase in H1 dynamics, since FRAP rates were similar whether or not RanBP7 was present in the reaction (Figure 1C). Importin beta, which also interacts with H1 and is similar to RanBP7 in size and charge, decondensed sperm chromatin to a lesser extent than RanBP7, while a mixture of 2 µM importin beta and 2 µM RanBP7 had comparable effects to 4 µM RanBP7 alone (Figure S1A). Thus, while RanBP7 and importin beta can decondense sperm chromatin and inhibit H1 in buffer, they cannot on their own reconstitute H1-chromatin binding dynamics.

### Cytoplasm Reduces H1 Binding and Increases Its Dynamics

**Figure 2:**
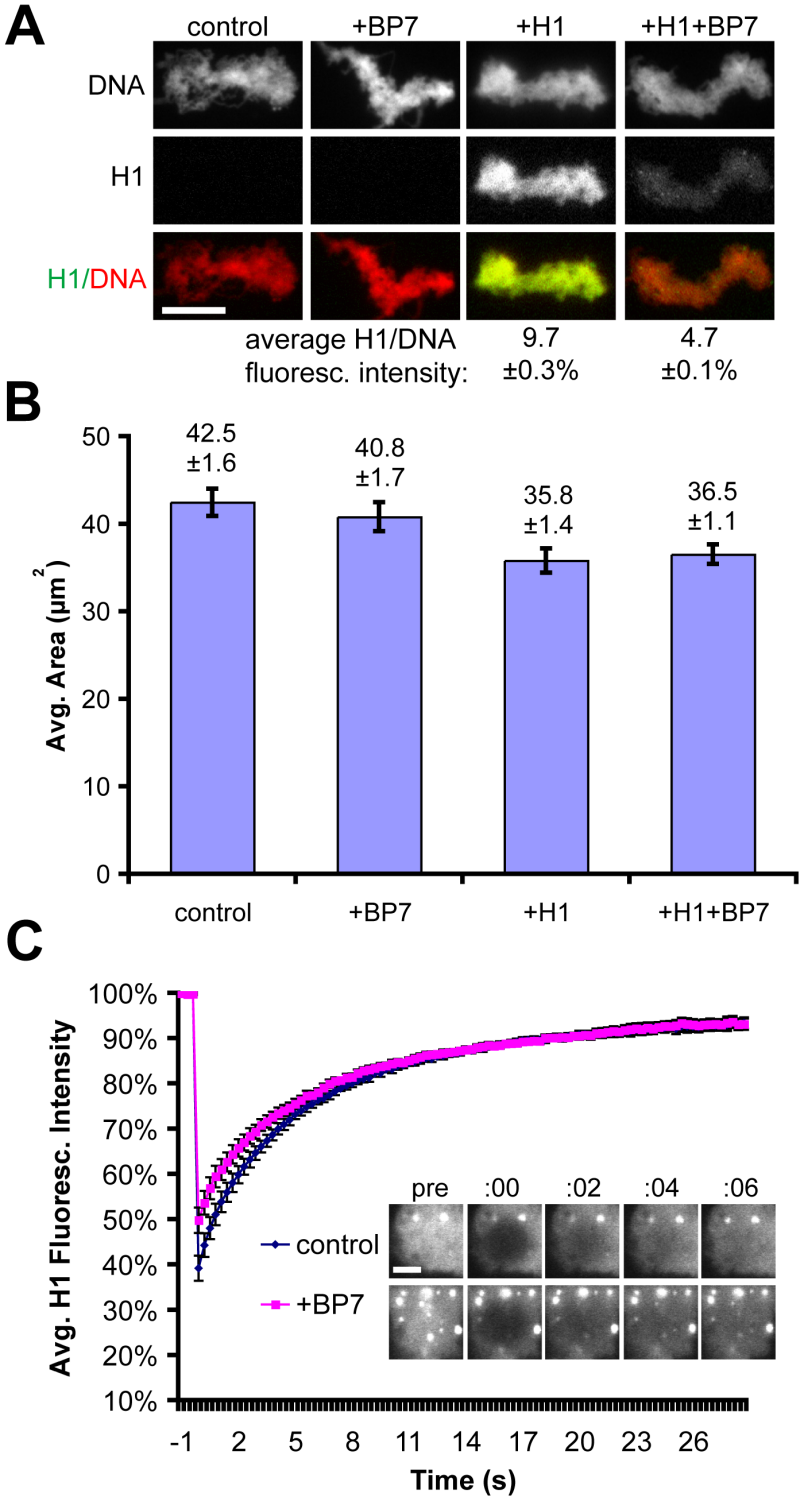
Effects of RanBP7 on H1 in Cytoplasm. (A) Fluorescence images and (B) area quantification of chromatin assembled in extracts with or without 1 µM H1-GFP and 4 µM RanBP7. In cytoplasm, adding RanBP7 does not greatly affect morphology but causes dissociation of H1 as measured by a reduction in fluorescence intensity. Average H1:DNA fluorescence intensity is shown below for conditions supplemented with H1. Scale bar, 10 µm. (C) Average FRAP curves (n≥7) and corresonding timelapse images of H1-GFP on chromatin in cytoplasm. H1-GFP recovers very rapidly and is not greatly affected by the addition of 4 µM recombinant RanBP7. H1-GFP signal was brightened in samples with RanBP7 relative to controls for visualization of the photobleaching and recovery. Photobleach occurs at time  = 0. Scale bar, 2 µm. All quantification is shown ± standard error.

To precisely measure the impact of cytoplasm on histone H1 chromatin binding and dynamics, we next performed exactly the same set of experiments in metaphase-arrested egg extracts instead of buffer. In this situation, the sperm nuclei are remodeled into larger clusters of unreplicated, compacted chromosomes. GFP-H1A bound mitotic chromatin more weakly in extract, with an H1:DNA intensity ratio approximately 25-fold lower than in buffer, while addition of RanBP7 further decreased the H1 signal by an additional 50% (Figure 2A). Unlike in buffer, addition of exogenous H1 or RanBP7 did not have dramatic effects on chromatin area (Figure 2B), possibly due to the presence of the embryonic linker histone H1M which binds chromatin tightly and does not interact strongly with RanBP7, as well as myriad other chromatin proteins present in the extract [22]. Also, in contrast with our observations of very slow H1 recovery after photobleaching in buffer, in cytoplasm H1 could not be photobleached to the same extent and indeed recovered remarkably quickly, with an apparent half-time of recovery of ∼7 seconds which was not enhanced by the addition of RanBP7 (Figure 2C and Movies S2 and S3). Addition of 4 µM importin beta to the extract had little effect on H1A-GFP levels on chromatin, while a mixture of 2 µM importin beta and 2 µM RanBP7 had intermediate effects, suggesting that heterodimer formation, which is required for H1 import into nuclei [25], may not be required for H1 inhibition in cytoplasm (Figure S1B). Altogether, these experiments demonstrate that while RanBP7 significantly reduces the ability of histone H1 to bind chromatin, it does not significantly impact H1 dynamics.

**Figure 3:**
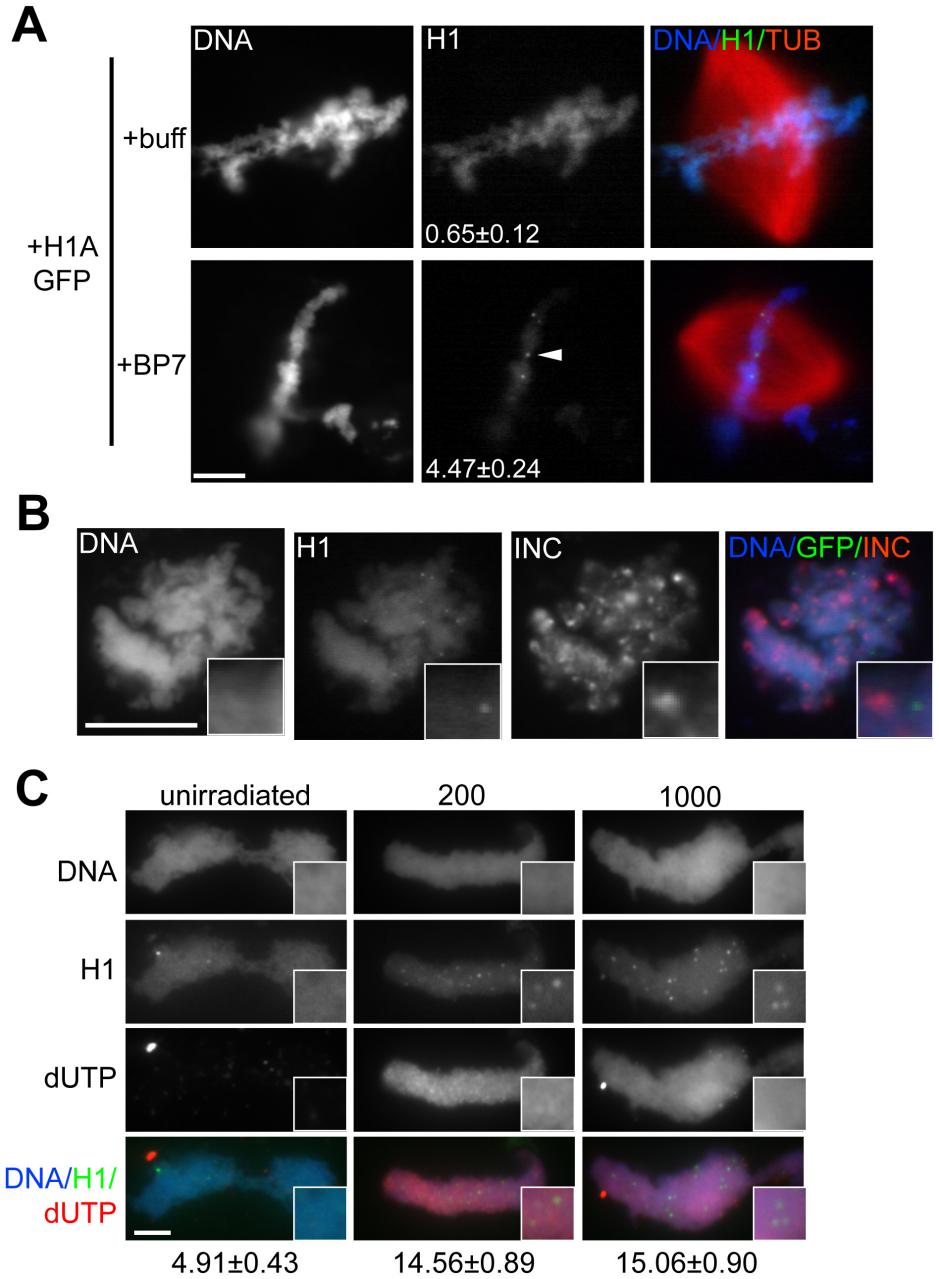
RanBP7 Reveals H1 Foci. (A) Identically-scaled fluorescence images of fixed metaphase spindles from CSF reactions supplemented with 1 µM H1A-GFP and 4 µM RanBP7 or buffer control (+buff). In the presence of RanBP7, H1A-GFP is reduced on chromatin and concentrates on chromatin in small foci (arrowhead). The number of foci per nucleus (average ± standard error, n>40) is shown in the H1A-GFP column. (B) H1A-GFP foci do not colocalize with the centromere marker INCENP (INC). INCENP localization was performed using replicated chromosomes, on which H1A-GFP foci were less obvious but still detectable (insets). (C) Immunofluorescence images of UV-irradiated or unirradiated sperm nuclei assembled into chromatin in metaphase extracts supplemented with H1A-GFP, RanBP7, and biotin-dUTP. Insets are provided and the number of foci per nucleus is shown below the column for each condition. Scale bars, 10 µm.

Interestingly, while the overall levels of H1 were reduced in the presence of RanBP7, bright foci of H1 were apparent on chromatin in this condition, with an average of 4–5 foci per nucleus within the epifluorescence focal plane (Figure 3A). Notably, centromeres did not co-localize with foci (Figure 3B). To test whether the foci might represent damaged DNA, sperm nuclei were pre-treated with ultraviolet irradiation. When UV-treated nuclei were incubated in metaphase cytoplasm, they incorporated labeled dUTP, a marker of DNA repair [28], and the number of H1 foci increased approximately 3-fold, however they did not completely overlap with the sites of DNA repair, suggesting a role in some but not all damaged loci (Figure 3C). The precise nature of these high-affinity H1 foci and their appearance in the presence of RanBP7 are interesting topics for further investigation.

### Effect of Cytoplasm on the H1M Isoform and Truncation Mutants

**Figure 4:**
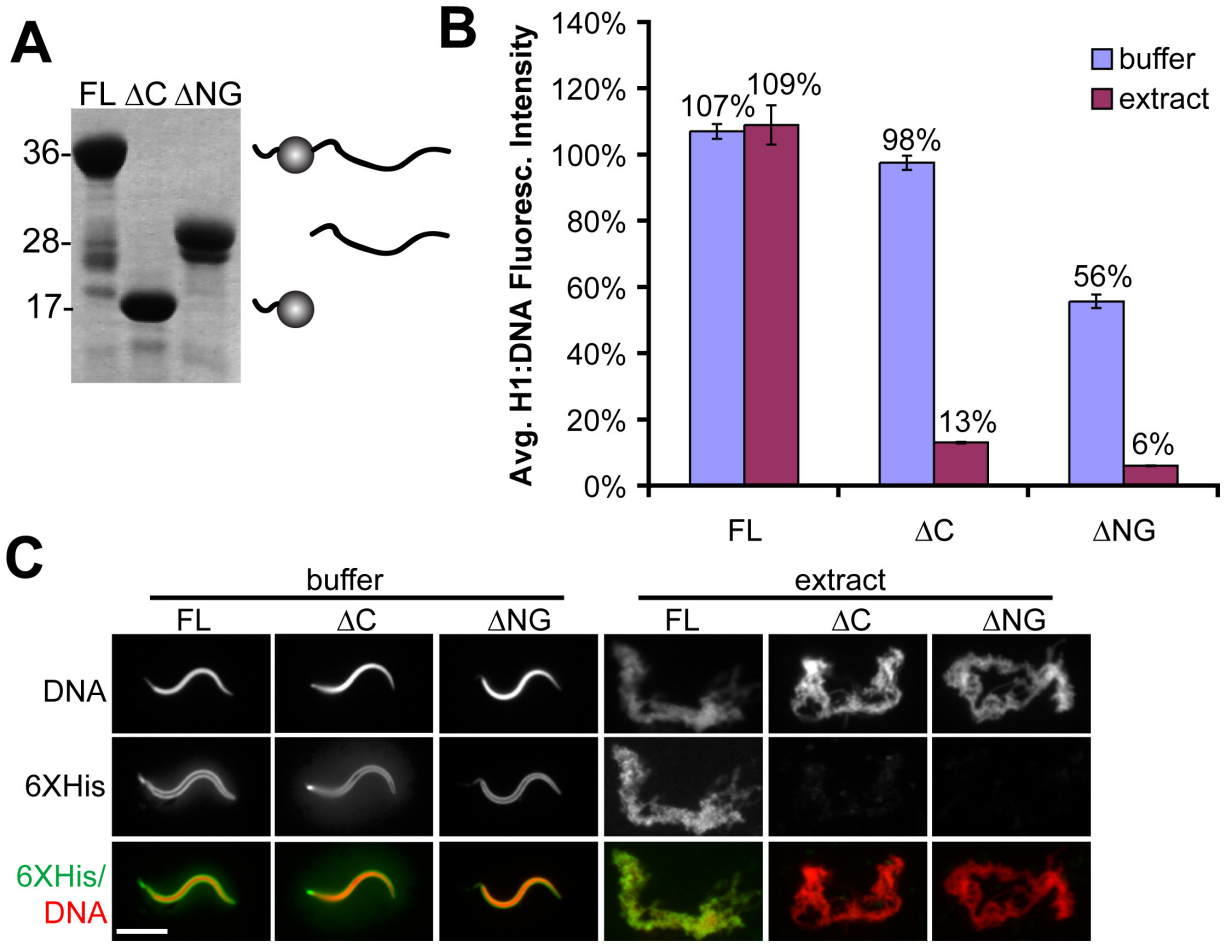
Effect of Cytoplasm on H1M and Domain Truncation Mutants. (A) Coomassie-stained gel of full-length (FL) H1M, amino-globular domains (ΔC), and C-terminal domain (ΔNG), with schematic of the proteins shown at right of the corresponding band. (B) Quantification of H1:DNA fluorescence intensity (average ± standard error) and (C) representative immunofluorescence images of sperm chromatin in buffer or extract supplemented with 1 µM H1M full-length or domains. In extract, full-length H1 localizes as efficiently as it does in buffer, while a sharp drop in localization intensity is observed for the domains. H1 was immunolocalized using the 6XHistidine tag (6XHis) common to all three constructs. For visualization purposes, DNA and H1 signal were brightened in the extract condition relative to buffer. Scale bar, 10 µm.

A recent functional comparison of somatic and embryonic H1 isoforms and revealed that the embryonic linker histone H1M, which is endogenous to the egg, does not interact strongly with importin beta or RanBP7, and binds more tightly to sperm chromatin than other H1 isoforms in egg extract [22]. The difference we observed in the binding affinity of full-length somatic H1 in buffer versus cytoplasm led us to inquire whether H1M might also function differently in these two environments. We purified recombinant H1M (Figure 4A), added it to buffer or cytoplasm at a concentration of 1 µM, and measured its binding to chromatin by immunofluorescence of the 6XHistidine tag. In contrast to somatic H1, H1M bound to chromatin very efficiently in cytoplasm, with an H1:DNA fluorescence intensity ratio identical to that observed in buffer (Figure 4B–C). The effects of cytoplasm on H1 affinity for chromatin are therefore isoform-specific.

The contribution of H1 domains to chromatin binding remains unclear, as truncation mutants can substitute for full-length H1 in some assays but also show reduced binding in living cells [7], [10], [11], [14], [15], [16], [17], [18]. We sought to determine the effect of cytoplasm on H1 truncation mutants by directly comparing their affinities for chromatin in buffer versus egg extract. We therefore expressed and purified two additional proteins: H1MΔC, comprising the short amino terminus of H1M plus the winged helix globular domain, and H1MΔNG, comprising the long, unstructured tail which is the remaining half of the protein (Figure 4A). We chose to examine truncation mutants of H1M rather than somatic H1 because the latter already has reduced affinity for chromatin in cytoplasm even in full-length form (Figure 2A; [22]). Full-length H1M or domain truncations were mixed with sperm at a concentration of 1 µM, and binding was assayed by immunofluorescence of the 6XHistidine tag common to all three proteins (Figure 4B–C). In buffer, all three proteins localized efficiently to sperm nuclei, although binding of H1MΔNG was reduced by 40–50% compared to full-length H1M or H1MΔC. However, in cytoplasm both H1MΔC and H1MΔNG were dramatically impaired in their ability to bind to sperm chromatin, achieving only ∼10% of the levels observed in buffer (Figure 4B–C). Similarly, in contrast to full-length H1M, neither on its own was able to rescue the effects of H1M immunodepletion from cytoplasm, which results in longer, thinner chromosomes ([21]; data not shown). H1MΔC and H1MΔNG obtained by specific proteolysis of full-length H1M gave identical results, and adding both domain truncations simultaneously did not result in cooperative binding (data not shown). The binding of individual H1M domain truncations to chromatin is therefore context-dependent, being relatively efficient in buffer but markedly impaired in cytoplasm when compared to full-length H1M.

**Figure 5:**
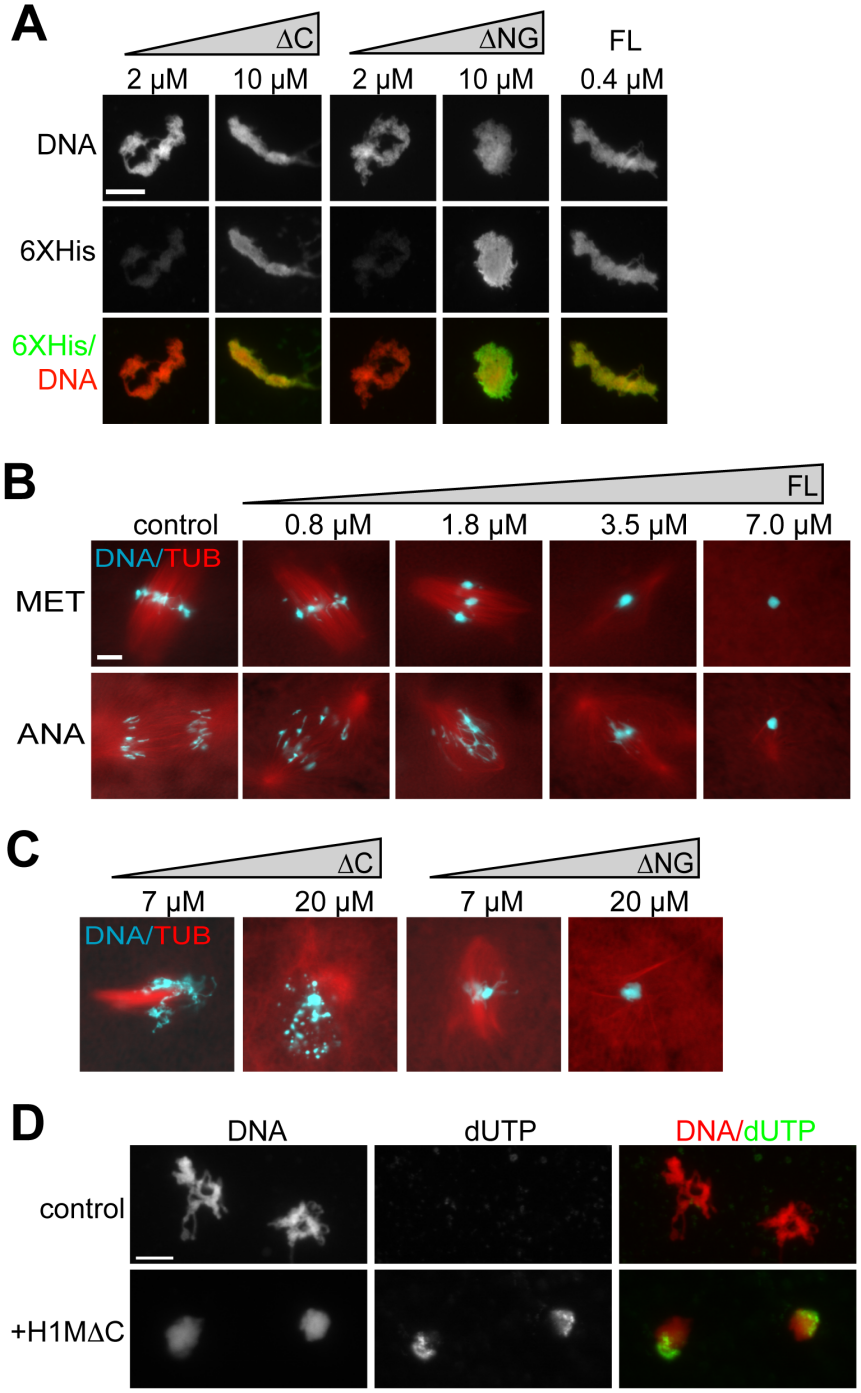
Overexpression Phenotypes of H1 Full Length and Domain Truncations in Extract. (A) Anti-6XHistidine immunofluorescence of chromosome clusters from reactions supplemented with increasing concentrations of amino-globular (ΔC) or C-terminal (ΔNG) H1M truncation mutants, or 0.4 µM H1M full-length (FL) as a control. Truncation mutants required much higher concentrations for efficient localization. (B) Squash fix of chromatin and rhodamine-labeled microtubules (TUB) in egg extracts supplemented with increasing concentrations of full-length H1M. Added at prophase, H1M compacts the condensing metaphase chromosomes (MET) into a single mass within aberrant spindles. Such hypercondensed chromosomes are unable to segregate during anaphase (ANA). (C) Metaphase reactions supplemented with amino-globular or C-terminal domains at high concentrations. H1MΔC causes chromosome fragmentation, while H1MΔNG causes mitotic chromosomes to compact into a single mass. (D) Representative, identically-scaled fluorescence images of sperm chromatin incubated in CSF egg extracts supplemented with biotin dUTP with or without 20 µM H1MΔC. Morphological chromatin fragmentation was less obvious using the immunofluorescence protocol, but was observed in squash samples from these reactions. Scale bars, 10 µM.

Since individual H1M domain truncations did not efficiently bind to chromatin in cytoplasm at concentrations of 1 µM, we added higher concentrations. 7–20 µM of H1MΔC or H1MΔNG did bind to chromatin, similar to much lower concentrations of full-length H1M (Figure 5A). We next investigated the functional ramifications of overexpressing H1M or its individual domain truncations in cytoplasm. When full-length H1M was added to cytoplasm reactions to concentrations ≥3.5 µM, individual mitotic chromosomes failed to resolve and instead packed tightly together, impairing spindle assembly and preventing chromosome segregation during anaphase (Figure 5B). Similar chromatin hypercompaction was observed when H1MΔNG was added at higher concentrations of 7–20 µM, while H1MΔC produced a different phenotype, causing aberrant chromatin fragmentation at these concentrations (Figure 5C). This aberrant fragmentation phenotype was accompanied by an increase in biotin-dUTP signal colocalizing with chromatin, indicating DNA damage (Figure 5D). H1MΔC overexpression caused no increase in caspase 3 activity relative to untreated extracts during the timecourse of DNA fragmentation (data not shown), suggesting that chromatin fragmentation was not caused by apoptosis, and indeed these extracts are known to be refractory to caspase activation [29]. These results show that individual H1M domain truncations can bind to chromatin in cytoplasm, but require significantly higher concentrations than full-length H1M, and distort mitotic chromatin morphology at these higher concentrations.

## Discussion

Our work reveals important differences between somatic H1 behavior *in vivo* and *in vitro.* Perhaps most striking is the failure of H1 to recover after photobleaching in the absence of cytoplasm (Figure 1C and 2C). Since it is well-established that H1 does not elute from purified chromatin in buffer at physiological salt concentrations [2], [16], static binding may be a general property of H1 in such systems, although we recognize the need to test this in other types of nuclei since sperm chromatin has a unique composition and limited nucleosome content [30]. ATP-depletion has been shown to slow H1 dynamics in living cells and in buffer [9], [31], but all of our experiments were performed in the presence of ATP and an energy-regenerating system. The very slow dynamics we observed in buffer were therefore not due to the lack of energy, but rather the lack of some cytoplasmic factor(s) promoting H1 dynamics, which may depend on ATP. Our results further demonstrate that while RanBP7 reduces somatic H1 binding to a similar degree in buffer and in cytoplasm, it cannot on its own reconstitute rapid H1 dynamics. A major question for further study is whether they could reconstitute dynamics in the presence of regulatory factors such as Ran, and if not then what other specific activities within cytoplasm, such as chromatin-remodeling ATPases, might result in the emergent property of dynamic H1 binding.

Reported half-times of recovery for H1 vary considerably, and our measurements in egg cytoplasm are most consistent with half-times on the order of seconds, not minutes [10]. This may reflect increased histone dynamics in embryonic cytoplasmic environments [32], or else result from the unusually high cytoplasmic:nuclear volume ratio in egg extracts (at a concentration of 1000 nuclei/µl, nuclei account for only ∼0.4% of the total reaction volume, assuming a nuclear diameter of 20 µm). The rapid off-rate of H1 in cytoplasm is likely connected with our observation that the intensity of somatic H1 on chromatin is only ∼10% of that in buffer. Interestingly, RanBP7 did not affect H1 dynamics but caused a ∼50% decrease in intensity of H1 on chromatin both in buffer and in cytoplasm (Figure 1A and 2A), suggesting that its interaction with H1 is not dramatically different between the two conditions. RanBP7 and importin beta are reported to act as a heterodimer [25], however supplementing extracts with 2 µM RanBP7 with 2 µM importin beta had effects similar to simply adding 4 µM RanBP7 (Figure S1A–B). The role of a heterodimer cannot be totally excluded, since there is an excess of importin beta already present in the egg extract, however no difference was observed between the heterodimer and the individual proteins in buffer either. We are inclined to conclude that while heterodimer formation is clearly required to transport somatic H1 across the nuclear envelope, that requirement is probably attributable to importin beta's specialized role during nuclear transport, where it must shield cargoes and quickly release them inside the nucleus to facilitate directional transport. In contrast, inhibition of somatic H1 binding to chromatin requires no specialized transport functions and can therefore proceed efficiently through a monomeric interactions with RanBP7, which may act directly as a competitive inhibitor. Our observation of distinct H1 foci in the RanBP7-treated condition is also interesting because linker histone staining is usually very homogeneous on chromatin. We believe it is unlikely that these foci represent aggregates of H1 caused by exogenous RanBP7, because on the contrary RanBP7 has been shown to stabilize H1 in cytoplasm and prevent its aggregation [25]. Based on the increase in foci number after UV irradiation and their appearance in live movies tethered to chromatin by very thin threads (Figure 3C and Movies S2 and S3), we suspect that they might represent unstructured DNA ends or double-stranded breaks. Such a localization pattern would be consistent with recent reports that somatic histone H1 co-purifies and stimulates complexes involved in non-homologous end joining and DNA double-stranded break repair [33], [34].

In contrast to somatic H1, when H1M was examined we observed identical H1:DNA fluorescence intensity ratios in buffer and cytoplasm (Figure 4B–C). This is consistent with our observation that H1M binds to mitotic chromatin in egg cytoplasm more efficiently than somatic H1 [22] and the observations of others that the reverse is true in buffer [23]. The high affinity of H1M for chromatin *in vivo* stands somewhat at odds with the reports of ours and others that GFP-tagged H1M expressed in cells has a rapid half-time of recovery after photobleaching, and indeed we have made similar observations in egg extract as well (B. Freedman and R. Heald, unpublished data). The simplest resolution for this apparent discrepancy may be that the GFP tag significantly reduces the affinity of H1M for chromatin *in vivo*, possibly by lowering its charge too far from the ideal (down from a pI of 10.11 to 9.56, comparable to the difference in charge between H1M and somatic H1).

Our results may also help explain superficially contradictory findings in the literature regarding the activity of H1 domains [7], [11], [15], [16]. In our experiments, cytoplasm has a much stronger inhibitory effect on individual domain truncations than on full-length H1M (Figure 4B–C). Therefore, although these domain truncations can function *in vitro* they cannot bind chromatin efficiently *in vivo*. While this behavior superficially resembles the situation for full-length somatic H1, the inhibitory factors affecting H1M domains are unlikely to be importin beta or RanBP7, since the full-length H1M isoform used for these experiments does not interact with these chaperones [22]. Whatever their identity, the combination of both amino-globular and unstructured carboxy-terminal H1 domains into one molecule appears to have a synergistic effect to overcome these inhibitory factors and enhance chromatin binding *in vivo*, which may explain why this domain structure is highly conserved among vertebrate linker histones. Cooperative interactions between the globular and carboxy-terminal domains have recently been estimated to enhance the binding affinity GFP-tagged somatic H1 by an order of magnitude or greater [35], [36], consistent with our own observations of ∼10-fold increase in fluorescence intensity of full length H1M relative to domain truncations. Although certain unicellular organisms possess linker histones that lack either the amino-globular or carboxyl terminal domains [17], [37], it is possible that such organisms either lack the cytoplasmic factors that inhibit H1 binding, or else overexpress these truncated linker histones to concentrations capable of overcoming such factors.

Despite decades of study, histone H1′s mechanism of action – how the globular domain and unstructured tail interact with chromatin and compact it *in vivo* - is still unclear. Our finding that the amino-globular and carboxyl terminal domains produce different overexpression phenotypes of fragmentation and compaction suggests that these two halves of the protein have different properties. We believe fragmentation caused by high levels of the amino-globular domain is due to a direct effect of the protein bound to DNA, leading to fragmentation upon chromosome condensation, and not because extracts are becoming apoptotic, since measured caspase 3 activity remained low. In contrast, the carboxyl terminal domain when overexpressed causes chromatin hypercompaction, possibly due to non-specific binding leading to chromatin aggregation. Thus, in addition to cooperative interactions which increase overall binding to chromatin, the amino-globular and carboxyl terminal domains of H1 likely contribute to separable functions of organization and compaction, both of which are required to stabilize chromatin without aggregation during dynamic remodeling processes.

## Materials and Methods

### Recombinant Proteins

H1MΔC (residues 1–131, including the unstructured amino terminus and the globular domain) and H1MΔNG (132–273) were identified by aligning the NCBI H1M sequence (gi:1587201) with the conserved H15 domain (cd00073) and cloned into vector pET30c (Novagen). Alternatively, a PreScission Protease site was encoded 3′ of the globular domain in the full-length H1M sequence using the QuikChange Mutagenesis Kit (Stratagene). H1 proteins and domains were purified from bacteria in PBS plus 500 mM NaCl and concentrated as described for H1A-GFP [22]. 6XHistidine-tagged human importin beta was purified from bacteria in PBS as described [38]. *X. laevis* RanBP7 (NCBI gi:148223036) was cloned as a TEV-cleavable His-tagged fusion, expressed in Rosetta cells in autoinduction media at 20 degrees Celsius, and purified in 25 mM HEPES pH 7.5, 10% glycerol, and 400 mM NaCl which was reduced after dialysis to 100 mM NaCl. Concentrations were determined by dilution series on a Coomassie-stained SDS-PAGE gel.

### Reactions with Sperm Nuclei

Demembranated sperm nuclei and CSF (cytostatic factor-arrested) low-speed egg extracts were prepared in XB and reacted at room temperature as described [19], [20]. For UV treatment, a Stratalinker was used to irradiate sperm with either 200 or 1000 J/m^2^. Caspase 3 activity was measured by the fluorescent reporter Ac-DEVD-pNA as described [29]. Proteins were added to extract prior to sperm addition or else immediately after sperm addition into XB buffer (100 mM KCl, 1 mM MgCl_2_, 0.1 mM CaCl_2_, 10 mM K-HEPES pH 7.7, 50 mM sucrose) supplemented with energy mix (3.75 mM creatinine phosphate, 0.5 mM Na_2_-ATP, 0.5 mM MgCl_2_, 50 µM EGTA). To label damaged DNA, biotin-16-dUTP (Roche) was added to metaphase reactions to a final concentration of 40 µM. 30 minutes after sperm addition, reactions were diluted 1∶10 into XB supplemented with 1 mM MgCl2, 5 mM EGTA, 0.25% Triton X-100, and 10% formaldehyde and processed for immunofluorescence as described [20], [21]. Antibodies used included mouse monoclonal anti-His (Clontech #631212) and rabbit polyclonal anti-hINCENP (Abcam #ab12183).

### Image Analysis and FRAP

Structures processed for immunofluorescence were imaged using identical exposures on an epifluorescence microscope (Olympus, model BX51) with CCD camera (Hamamatsu, model C4742-98), shutter controller (Sutter Instrument Co., model Lambda 10-2), and the free µManager plugin for ImageJ (www.micro-manager.org) using a 40X dry objective (Olympus, N.A. 0.75). Image files were then subjected to automated structure identification and morphometric/intensity colocalization analysis using free CellProfiler software (www.cellprofiler.org). For FRAP, 2 µl of reaction was spotted onto a PEG-coated slide, overlaid with a 12 mm circular coverglass, and imaged every 300 ns with a 60X oil objective on a Zeiss Aviovert200M confocal microscope running LSM software. A 3 second photobleach at 100% power for the Argon/488 laser was applied to a 1–2 µm diameter circle on individual metaphase plates. Stacks were aligned and curve-fitted using the FRAP Profiler plugin for ImageJ.

## Supporting Information

Figure S1 Effect of RanBP7/Importin beta on Sperm Chromatin. (A) Average areas of sperm pronuclei in buffer supplemented with 4 μM RanBP7, 4 μM importin beta (IB) or 2 μM of each. Addition of H1A-GFP rescues the size increase. (B) Identically-scaled fluorescence images of H1A-GFP (1 μM) and rhodamine-labeled tubulin in CSF reactions supplemented with 4 μM importin beta, 4 μM RanBP7, 2 μM of each, or buffer control. Average H1A-GFP:DNA intensities are shown below each column. Scale bar, 10 μm.
(1.43 MB TIF)

Movie S1 FRAP of H1A-GFP on sperm chromatin in buffer (elapsed time  =  30 seconds).
(6.56 MB AVI)

Movie S2 FRAP of H1A-GFP on sperm chromatin in egg extract (elapsed time  =  30 seconds).
(6.56 MB AVI)

Movie S3 FRAP of H1A-GFP on sperm chromatin in egg extract supplemented with RanBP7 (elapsed time  =  30 seconds).
(6.56 MB AVI)
