# Supplementary figures and images for: Xenopus Egg Extracts Increase Dynamics of Histone H1 on Sperm Chromatin
Source: PLoS One. 2010 Sep 29;5(9):e13111. doi: 10.1371/journal.pone.0013111 (PMC2947519; doi:10.1371/journal.pone.0013111)

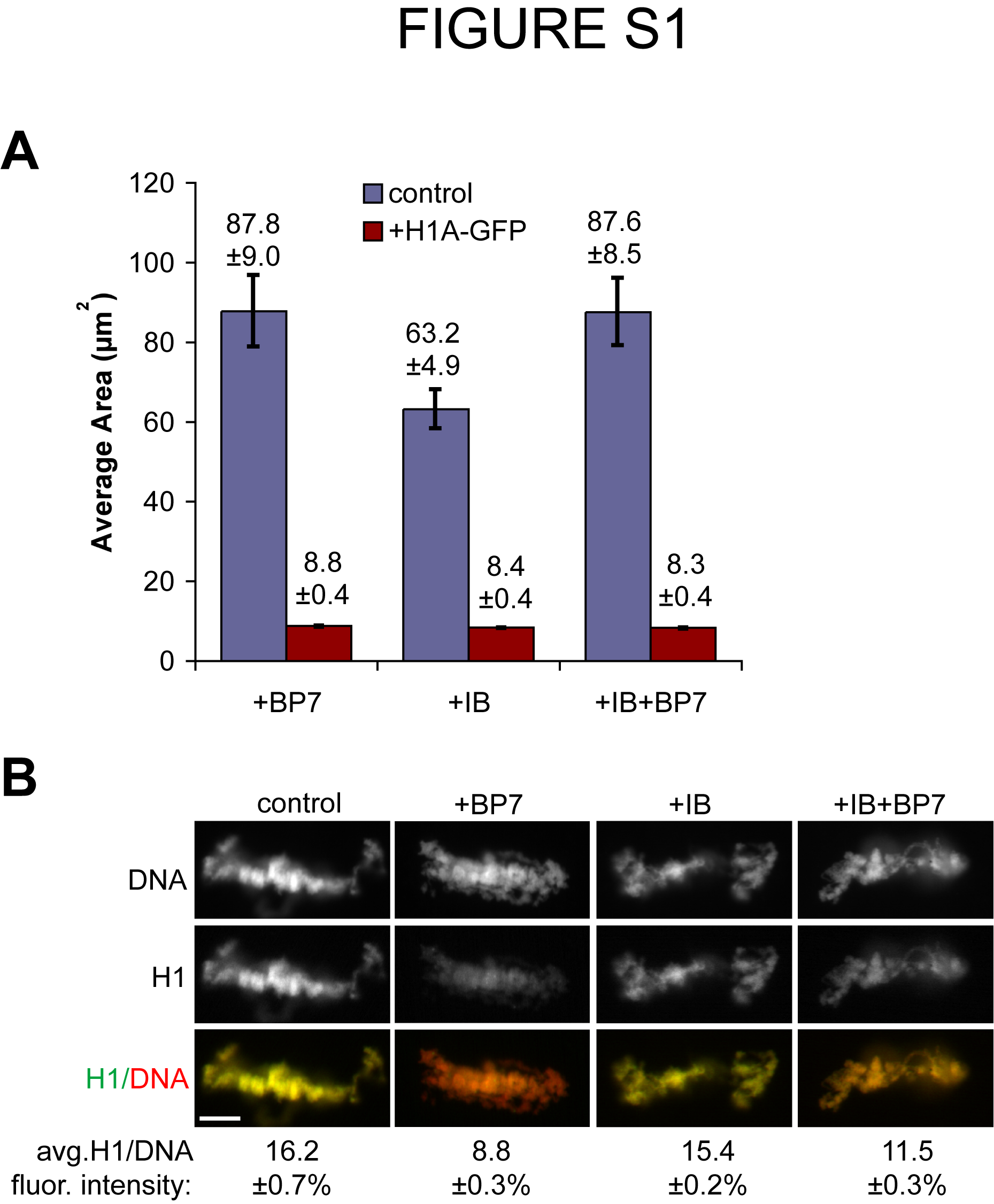

Supplement: Figure S1 — Effect of RanBP7/Importin beta on Sperm Chromatin. (A) Average areas of sperm pronuclei in buffer supplemented with 4 μM RanBP7, 4 μM importin beta (IB) or 2 μM of each. Addition of H1A-GFP rescues the size increase. (B) Identically-scaled fluorescence images of H1A-GFP (1 μM) and rhodamine-labeled tubulin in CSF reactions supplemented with 4 μM importin beta, 4 μM RanBP7, 2 μM of each, or buffer control. Average H1A-GFP:DNA intensities are shown below each column. Scale bar, 10 μm. (1.43 MB TIF) [file pone.0013111.s001.tif]
